# Association between platelet count and the risk and progression of hand, foot, and mouth disease among children

**DOI:** 10.6061/clinics/2020/e1619

**Published:** 2020-05-11

**Authors:** Li Miao, Yongjuan Liu, Peiliang Luo, Song Mao, Jiansheng Liu, Siguang Lu

**Affiliations:** I Department of Pediatrics, The First Affiliated Hospital of Kangda College of Nanjing Medical University /, The First People's Hospital of Lianyungang, Lianyungang, China; II Department of Pediatric Nephrology, Lianyungang Children's Hospital, Lianyungang, China; III Department of Central Laboratory, The First Affiliated Hospital of Kangda College of Nanjing Medical University /, The First People's Hospital of Lianyungang, Lianyungang, China; IV Department of Pediatrics, Shanghai Jiao Tong University Affiliated Sixth People's Hospital, Shanghai, China

**Keywords:** Platelet, Risk, Progression, Hand, Foot, Mouth Disease

## Abstract

**OBJECTIVE:**

We aimed to evaluate the association between platelet (PLT) count and the risk and progression of hand, foot, and mouth disease (HFMD).

**METHODS:**

In total, 122 HFMD patients and 40 healthy controls were enrolled in the study. The differences between variables among the different subgroups were compared. Logistic regression analyses were performed to assess the relationship between various parameters and HFMD risk/progression. Sensitivity analysis was conducted by detecting the trend of the association between PLT count quartiles and HFMD risk/progression. A generalized additive model was used to identify the nonlinear relationship between PLT count and HFMD risk/progression. The relationship between gender and PLT count as well as the risk/progression of HFMD was detected using a stratified logistic regression model.

**RESULTS:**

Significant differences were observed in terms of age, male/female ratio, white blood cell (WBC) count, and PLT count between patients with stage I-II, III-IV HFMD and healthy controls. Moreover, the alanine aminotransferase and magnesium levels between patients with stage I-II and III-IV HFMD significantly differed. Moreover, a significant difference was noted in the male/female ratio among the different PLT groups. The group with a low PLT count had a lower risk of HFMD progression than the group with a high PLT count (Q4) (*p*=0.039). Lower age, male gender, and WBC count were found to be associated with HFMD risk. Meanwhile, PLT count was correlated to HFMD progression. The sensitivity analysis yielded a similar result using the minimally adjusted model (*p* for trend=0.037), and minimal changes were observed using the crude and fully adjusted model (*p* for trend=0.054; 0.090). A significant nonlinear relationship was observed between PLT count and HFMD progression after adjusting for age, gender, and WBC (*p*=0.039).

**CONCLUSIONS:**

PLT was independently associated with HFMD progression in a nonlinear manner.

## INTRODUCTION

Hand, foot, and mouth disease (HFMD) is a common syndrome mainly caused by intestinal viruses of the picornaviridae family 1. HFMD usually affects children younger than 5 years 2. The most common clinical symptoms are ulcers and rashes on certain body parts 3. Most patients with mild HFMD can recover spontaneously. However, about 1.1% of individuals in China develop severe HFMD and present with serious clinical complications, such as encephalitis, aseptic meningitis, and even death, during progression 4,5. Antiviral therapy and vaccination against certain enterovirus have been the primary methods used for treating and preventing HFMD 6,7. However, in severe cases, patients are resistant to the traditional therapy and had a poor prognosis. Due to its potential danger, HFMD has become an important health concern among children worldwide. Thus, early prevention and therapy of HFMD may be imperative.

Recently, several studies 8-10 have focused on the risk factors of severe HFMD. Some pathogens, including enterovirus 71, were found to be responsible for the progression of HFMD 11. Moreover, inflammation was implicated in the development of HFMD 12. Meteorological and geographical factors were closely associated with the incidence of HFMD 13. However, there are some limitations in the management of such condition. For example, several laboratory parameter tests are not available in primary medical institutions. On the other hand, a high number of children present with herpangina and HFMD in suburban districts during the epidemic season. Therefore, the identification of low-cost and easily available alternative biomarkers for HFMD risk and progression is of great clinical significance.

Platelet (PLT) is an important component of the blood, and it plays an important role in several physiological and pathological processes, such as coagulation, thrombosis, and inflammation 14. PLT count is associated with inflammatory diseases. Inflammation correlated with endothelial dysfunction is a cause of organ failure and is associated with PLT activation and consumption 15. In addition, a relationship was observed between changes in PLT count and the prognosis of critically ill patients 16-18. Inflammatory and thrombotic conditions may cause changes in PLT size 19. Larger PLTs are more reactive than smaller ones as they can more easily release chemical mediators in response to various stimuli. Hence, alterations in PLT count are associated with morbidity and mortality in patients with various diseases. Moreover, PLT is easily available as it is a parameter of complete blood count test, which is performed using the automatic analyzer. Thus, it can be generalized and applied in primary medical institutions.

To identify the possible role of PLT in HFMD, we evaluated the association between PLT and HFMD risk and progression. HFMD was classified into four clinical stages based on the involvement of injured organs and disease severity. Meanwhile, the relationship between other clinical/laboratory parameters and HFMD as well as the role of other variables on the association between PLT and HFMD were analyzed.

## MATERIAL AND METHODS

### Patient population

The patients diagnosed with HFMD at First People's Hospital of Lianyungang between January 2016 and April 2018 were screened. Nearly 600 patients visit the outpatient department daily. Patients who were diagnosed with HFMD at presentation were enrolled in the current study. HFMD was diagnosed according to the World Health Organization criteria for HFMD 20. The patients with HFMD were divided into four groups based on clinical stages: clinical stage I (no involvement of other organs), stage II (involvement of the nervous system), stage III (early cardiopulmonary failure), and stage IV (cardiopulmonary failure). However, patients with signs and symptoms not consistent with the diagnostic criteria and those who were immunosuppressed were excluded. This retrospective study was conducted according to the Declaration of Helsinki. Moreover, informed consent was obtained from the guardians of all participants.

### Data collection

We obtained data about the clinical and laboratory parameters of the patients from the medical records. Demographic and clinical data, such as age and gender, were reviewed respectively. Laboratory data included white blood cell (WBC) count and hemoglobulin (HB), PLT, alanine aminotransferase (ALT), aspartate aminotransferase (AST), albumin (ALB), serum creatinine (Scr), calcium (Ca), phosphorus (P), and magnesium (Mg) levels. All parameters were assessed during the first day after the admission of the patients to the hospital. The data of the controls, including age, gender, WBC count, and PLT count, were obtained. PLT count was also expressed in quartiles.

### Statistical analysis

Continuous variables were expressed as mean/standard deviation of the normal distribution or median/quartile in the skewed distribution. Meanwhile, categorical variables were presented as frequency. One-way analysis of variance and the Kruskal–Wallis H test were used to detect the differences in continuous variables. The chi-square test was utilized to identify the differences in categorical variables. Logistic regression analyses were used to assess the relationship between various clinical and laboratory parameters and HFMD risk/progression. The association between PLT count and HFMD risk/progression was assessed via a logistic regression analysis using the crude, minimally adjusted, and fully adjusted models. Moreover, a sensitivity analysis was performed by detecting the trend of the association between PLT count quartiles and HFMD risk/progression. A generalized additive model was used to identify the nonlinear relationship between PLT count and HFMD risk/progression. A subgroup analysis in terms of sex of the patient was conducted using the stratified logistic regression model. The likelihood ratio test was used to detect the interaction between the subgroups. Statistical analyses were conducted using the software R and EmpowerStats (http://www.empowerstats.com, X&Y Solutions, Inc., Boston, MA). A *p* value <5% was considered statistically significant.

## RESULTS

### Baseline characteristics of the participants

A total of 122 HFMD patients and 40 controls were enrolled in our study (Table 1). There were 104 and 18 patients with stage I-II (n=40, stage I; n=64, stage II) and III-IV HFMD, respectively. The median ages of the patients with stage I-II HMFD, those with stage III-IV HFMD, and controls were 1.83, 1.87, and 6, respectively. Significant differences were observed in terms of age, male/female ratio, WBC count, and PLT count among patients with stage I-II and III-IV HFMD and controls. Moreover, the ALT and Mg levels between patients with stage I-II and those with III-IV HFMD significantly differed. No statistically significant differences were observed in terms of age, WBC count, and HB, ALT, AST, Scr, Ca, P, and Mg levels among the different PLT groups (Table 2). The male/female ratio significantly differed among the PLT groups (Table 2). The group with a low PLT count (Q1) had a lower risk of HFMD progression than the group with a high PLT count (Q4) (*p*=0.039) (Table 2).

### Univariate logistic regression analysis

The results of the univariate logistic regression analyses are shown in Table 3. WBC count was positively associated with HFMD risk (95% CI: 1.035-1.346, *p*=0.014), indicating that higher level of WBC count increased the risk of HFMD susceptibility. Lower age and male gender were also found to be associated with HFMD risk. However, HB, PLT, ALB, ALT, AST, Scr, Ca, P, and Mg levels were not associated with HFMD risk, and a relationship was not observed between age, male gender, WBC count, and HB, ALB, ALT, AST, Scr, Ca, and *p* levels as well as HFMD progression.

### Relationship between PLT count and HFMD risk and progression

We used the univariate logistic regression model to evaluate the association between PLT count and HFMD. The non-adjusted and adjusted models are shown in Table 4.

In the crude model, PLT count was not associated with HFMD risk. Moreover, similar results were obtained using the minimally adjusted and fully adjusted models. In the sensitivity analysis, PLT was considered a categorical variable (quartile), and the same trend was observed. PLT count was found to be associated with HFMD progression regardless whether the crude, minimally, and fully adjusted model was used. Sensitivity analysis yielded a similar result using the minimally adjusted model, and only minimal changes were observed using the crude and fully adjusted models.

### Analyses of nonlinear relationships

**Figure 1:**
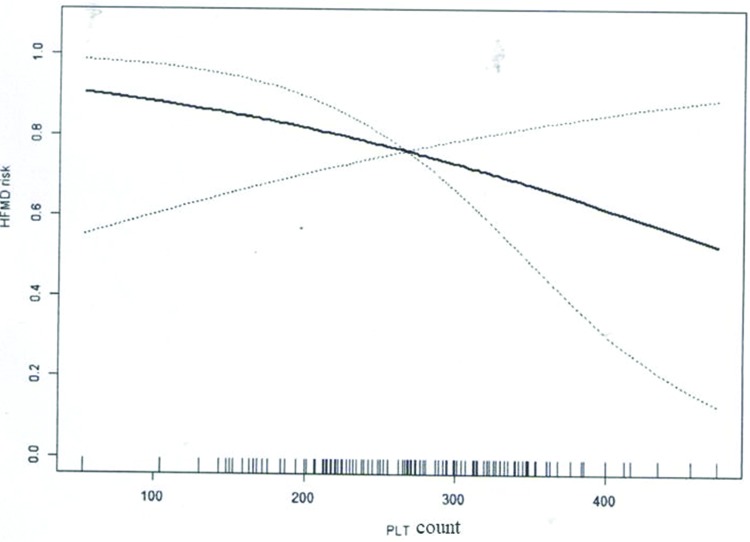
Nonlinear association between PLT count and HFMD risk.

**Figure 2:**
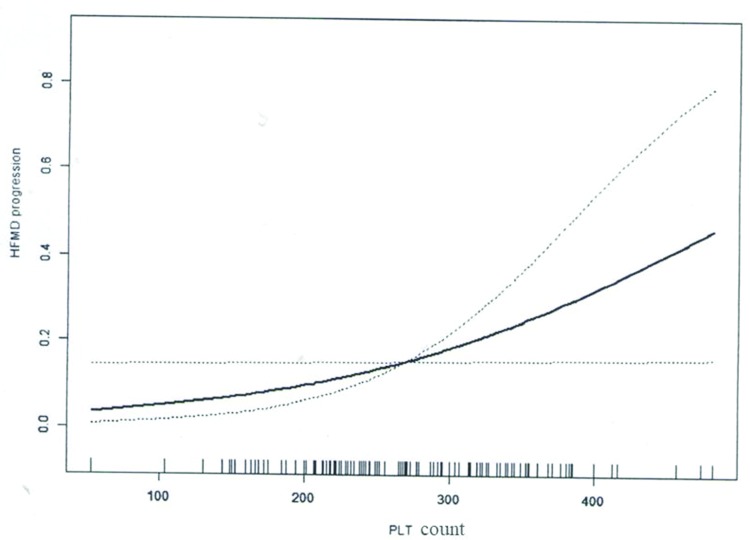
Nonlinear association between PLT count and HFMD progression.

With the use of PLT as a continuous variable, the nonlinear relationship between PLT count and HFMD risk/progression was assessed. Results showed that PLT count was not associated with HFMD risk after adjusting for age, gender, and WBC count based on the nonlinear relationship analysis (Table 5, Figure 1). Notably, a significant nonlinear relationship was noted between PLT count and HFMD progression after adjusting for age, gender, and WBC (Table 5, Figure 2).

### Results of the subgroup analyses

As shown in Table 6, the relationship between PLT count/quartiles and gender as well as HFMD risk and progression was not significant.

## DISCUSSION

Increasingly attention has been paid to the prevention and monitoring of HFMD. Outbreaks of HFMD commonly occur worldwide 21. The identification of a low-cost, easily available predictor for HFMD risk and progression is effective for the prevention of HFMD. This study first investigated the association between PLT count and the risk and progression of HFMD. Our investigation showed that a significant, independent, and nonlinear relationship existed between PLT count and HFMD progression. Moreover, PLT can be an alternative, low-cost biomarker for HFMD progression.

Several mechanisms may account for our findings. First, platelets promote the transfer of progenitor cells and leukocytes to the inflammation sites and release of several substances that mediate inflammation 22. Inflammation plays an important role in the development of HFMD. Systemic inflammatory response may play a role in the pathogenesis of HFMD 23. Enterovirus, which causes HFMD, significantly increases the release of circulating inflammatory mediators 23. Interleukin (IL)-27 is associated with early cardiopulmonary failure in enterovirus-induced HFMD 24. Cytokines and chemokines play a role in the development of HFMD 25. Moreover, patients with mild HFMD but without nervous system complications had increased levels of serum inflammatory cytokines 25. The abovementioned data support the notion that PLT may increase the susceptibility to HFMD by promoting inflammation. Studies have shown that platelets contribute to the risk of sepsis and acute lung injury 26. Moreover, they play an important role in trans-cellular metabolism, leading to the production of pro-inflammatory and anti-inflammatory molecules 27. An inflamed endothelium was found to be associated with leukocytes, which can roll on the template of adherent platelets and can migrate through the platelets 28. Second, platelet size can affect these functions. Mean PLT volume is the most commonly used marker in evaluating the changes in platelet function and activation. Platelets levels increase in high-grade inflammation, resulting in the decreased level of mean PLT volume due to the migration of large reactive platelets to inflammatory sites and the consumption of these platelets 29. Finally, HFMD is commonly caused by multiple viruses, including enterovirus 30, which may affect the PLT count. Severe HFMD is often complicated by organ damage, which may exacerbate inflammatory response, and this phenomenon is associated with platelet activation. Increased PLT count may be an early warning sign of severe HFMD, thereby indicating that a more aggressive treatment may be required.

Our findings also give rise to two questions. First, the in-depth mechanism behind the role of PLT in the development of HFMD must be further investigated. In addition to inflammation, other factors may also be involved in the pathogenesis of HFMD. Second, multiple pathogens can induce the onset of HFMD. Whether a close relationship exists between PLT count and the infectious pathogens of HFMD remains unclear. Previous studies have investigated the risk factors for HFMD progression. Chen et al. 23 have shown that IL-6, IL-10, and IL-13 were associated with the development of HFMD in children. David Lee et al. 13 have presented that weather conditions and geographic location play a role in the occurrence of HFMD epidemics. Liu et al. 31 have revealed that a moderately warm environment promotes the transmission of HFMD viruses, whereas particularly cold and hot climate conditions inhibit their transmission. Liu et al. 32 have reported that blisters and constipation are potential warming signs, and front-line clinicians must manage the sudden increase in the number of children diagnosed with mild HFMD during a pandemic. Previous findings indicated that the incidence and progression of HFMD are associated with environmental factors, which can affect the risk of inflammation. PLT may affect HFMD progression by regulating inflammation. Our findings were consistent with the abovementioned data, and PLT was found to be correlated the severity of HFMD. Interestingly, PLT was not associated with the risk of stage I-II HFMD, indicating that PLT was not involved in the development of early-stage HFMD. Notably, in the epidemic of HFMD or in susceptible cases, the changes in PLT count could not reflect the risk of HFMD. Meanwhile, in HFMD cases, PLT count reflects the severity of HFMD.

This study had several limitations that should be considered. First, its retrospective study design might have led to recall bias. However, we obtained comparatively reliable data. Second, the correlation analysis between PLT count and prognosis was not assessed due to the lack of prognostic data. Finally, the limited number of participants reduced the statistical power. Hence, studies with a larger sample size should be conducted in the future.

In conclusion, PLT count was independently associated with HFMD progression in a nonlinear manner. Thus, it can be used as an alternative, low-cost biomarker for HFMD progression.

## AUTHOR CONTRIBUTIONS

Miao L and Liu Y performed the data collection. Liu J and Lu S designed the study. Mao S and Luo P were responsible for the data analyses and manuscript writing.

## Figures and Tables

**Table 1:** Baseline characteristics of the participants in terms of disease state.

	Patients with stage I-II HFMD (n=104)	Patients with stage III-IV HFMD (n=18)	Controls (n=40)	*p*-value
Age (years)	1.83 (1.31, 2)	1.87 (1.21, 2.75)	6 (4, 8.25)	<10-4
Gender (male/female)	76/28	15/3	20/20	0.01
WBC count (109/L)	11.29 (9.43, 14.57)	12.95 (10.71, 17.92)	8.75 (6.95, 10.98)	0.001
HB level (g/L)	119.5 (114, 123)	118 (109, 125)	--	0.86
PLT count (109/L)	261.23±77.02	309.06±74.49	283.75±55.86	0.044
ALT level (u/L)	16 (12, 21)	14.5 (12, 19.5)	--	0.046
AST level (u/L)	31 (26, 41)	27 (25, 38)	--	0.14
ALB level (g/L)	45.6 (43.2, 47.7)	45.75 (43.58, 48.83)	--	0.99
Scr level (umol/L)	21.45 (17.38, 24.9)	20 (17.8, 22.8)	--	0.53
Ca level (mmol/L)	2.48±0.10	2.46±0.13	--	0.39
P level (mmol/L)	1.76±0.21	1.75±0.22	--	0.79
Mg level (mmol/L)	0.90±0.09	0.85±0.08	--	0.024

HFMD: hand, foot, and mouth disease, WBC: white blood cell, HB: hemoglobulin, PLT: platelet, ALT: alanine aminotransferase, AST: aspartate aminotransferase, ALB: albumin, Scr: serum creatinine, Ca: calcium, P: phosphorus, Mg: magnesium.

**Table 2:** Baseline characteristics of the participants in terms of PLT quartiles.

	Q1 (40)	Q2 (38)	Q3 (40)	Q4 (40)	*p*-value
Age (years)	2.0 (1.33, 3)	2.5 (1.83, 5.75)	2 (1.33, 4.0)	2 (1.65, 4.0)	0.80
Gender (male/female)	28/12	33/5	20/20	26/14	0.006
WBC count (109/L)	10.41 (9.24, 12.78)	10.77 (8.09, 12.67)	10.32 (8.02, 14.62)	12.2 (10.26, 14.80)	0.27
HB level (g/L)	118.5 (111.75, 123.5)	119.5 (113.5, 125.5)	119 (111, 122)	119 (116, 122.25)	0.78
ALT level (u/L)	14 (11, 21)	16 (13, 20.0)	16 (13, 22.2)	16 (12, 20.2)	0.729
AST level (u/L)	31 (26, 39)	30 (27, 39)	31.5 (25.9, 40.2)	31 (25, 41)	0.923
ALB level (g/L)	43.9 (42.6, 46.1)	45.4 (43.9, 47.3)	46.3 (44.2, 48)	45.7 (43.4, 48.3)	0.133
Scr level (umol/L)	22.4 (16.9, 23.8)	22.8 (19.3, 25.7)	21.1 (16.9, 24.3)	20.5 (17.8, 24.8)	0.621
Ca level (mmol/L)	2.46±0.11	2.46±0.09	2.48±0.12	2.50±0.11	0.419
P level (mmol/L)	1.72±0.20	1.71±0.17	1.79±0.21	1.78±0.23	0.202
Mg level (mmol/L)	0.89±0.10	0.89±0.09	0.91±0.10	0.88±0.09	0.647
HFMD				Q4 *vs* Q1	0.084
None	6	12	11	11	
Stage I-II	32	22	24	22	
HFMD				Q4 *vs* Q1	0.039
Stage I-II	32	22	24	22	
Stage III-IV	2	4	5	7	

HFMD: hand, foot, and mouth disease, WBC: white blood cell, HB: hemoglobulin, PLT: platelet, ALT: alanine aminotransferase, AST: aspartate aminotransferase, ALB: albumin, Scr: serum creatinine, Ca: calcium, P: phosphorus, Mg: magnesium.

**Table 3:** Univariate analysis results (HFMD stage I-II *versus* control; *HFMD III-IV *versus* HFMD stage I-II).

Index	Effect size (**β**)		95% CI		*p*-value	
Age	0.233	*1.234	0.140-0.388	*0.829-1.835	<10-4	*0.299
Gender						
Female	Reference: 1		−		−	
Male	2.714	*1.842	1.274-5.782	*0.495-6.848	0.0096	*0.362
WBC count	1.180	*1.1093	1.035-1.346	*0.995-1.237	0.014	*0.062
HB level	−0.001	*0.995	−0.001-0.001	*0.943-1.049	0.999	*0.855
PLT count	0.996	*1.008	0.990-1.0008	*1.001-1.015	0.097	*0.020
ALB level	0.999	*0.999	0.00001-1.0001	*0.868-1.149	0.999	*0.985
ALT level	0.001	*0.973	−0.001-0.001	*0.914-1.036	0.999	*0.392
AST level	−0.001	*0.976	−0.001-0.001	*0.933-1.021	0.999	*0.289
Scr level	0.001	*0.978	−0.001-0.001	*0.9001-1.062	0.999	*0.597
Ca level	−0.001	*0.126	−0.001-0.001	*0.0012-13.103	0.999	*0.382
P lebel	0.001	*0.725	−0.001-0.001	*0.067-7.842	0.999	*0.791
Mg level	−0.001	*0.002	−0.001-0.001	*0.00001-0.505	0.998	*0.028

HFMD: hand, foot, and mouth disease, WBC: white blood cell, HB: hemoglobulin, PLT: platelet, ALB: albumin, ALT: alanine aminotransferase, AST: aspartate aminotransferase, Scr: serum creatinine, Ca: calcium, P: phosphorus, Mg: magnesium.

**Table 4:** Relationship between PLT and HFMD risk and progression in the different models.

Index	Crude model	Minimally adjusted model	Fully adjusted model
PLT count	#0.996 (0.990-1.001), 0.097	#0.993 (0.984-1.003), 0.172	#0.999 (0.998-1.0003), 0.999
	*1.008 (1.001-1.015), 0.02	*1.008 (1.002-1.015), 0.016	*1.009 (1.001-1.018), 0.036
PLT quartile			
Q1	#Reference	#Reference	#Reference
	*Reference	*Reference	*Reference
Q2	#0.345 (0.112-1.054), 0.062	#0.672 (0.071-6.347), 0.729	#0.999 (0.00001-1.0004), 0.999
	*2.909 (0.489-17.287), 0.240	*2.585 (0.429-15.590), 0.300	*3.021 (0.393-23.213), 0.288
Q3	#0.409 (0.133-1.262), 0.119	#0.247 (0.029-2.094), 0.199	#0.999 (0.00001-1.0003), 0.999
	*3.333 (0.595-18.674), 0.171	*4.019 (0.691-23.391), 0.122	*2.728 (0.317-23.456), 0.361
Q4	#0.375 (0.121-1.165), 0.105	#0.237 (0.029-1.956), 0.181	#0.999 (0.00001-1.0005), 0.999
	*5.091(0.966-26.844), 0.055	*5.337 (1.002-28.419), 0.049	*6.585 (0.822-52.724), 0.076
P for trend	#0.136	#0.122	#0.999
	*0.054	*0.037	*0.090

HFMD: hand, foot, and mouth disease, PLT: platelet, crude model: adjusted for none, minimally adjusted model: adjusted for age and gender, fully adjusted model: adjusted for age, gender, WBC count and HB, ALB, ALT, AST, Scr, Ca, P, and Mg levels;

# relationship between PLT count and HFMD risk

* relationship between PLT count and HFMD progression.

**Table 5:** Nonlinear association between PLT count and HFMD risk/progression.

Index	Effect size (**β**)	95% CI	*p*-value
HFMD risk	961.27	19.33-47802.25	0.296
HFMD progression	0.016	0.0016-0.163	0.039

HFMD: hand, foot, and mouth disease, PLT: platelet.

**Table 6:** Role of gender on the association between PLT count and HFMD risk/progression.

Index	Effect size (**β**)	95% CI	*p* for interaction
#HFMD risk			
Male	0.997	0.987-1.007	0.089
Female	0.973	0.947-0.999	
*HFMD risk			
Male	0.996	0.981-1.012	0.290
Female	0.982	0.958-1.006	
#HFMD progression			
Male	1.007	0.999-1.014	0.688
Female	1.011	0.993-1.029	
*HFMD progression			
Male	1.010	1.001-1.021	0.198
Female	0.994	0.973-1.016	

HFMD: hand, foot, and mouth disease, PLT: platelet

# PLT count and HFMD

* PLT Q4 and HFMD.
